# Patterned Hippocampal Stimulation Facilitates Memory in Patients With a History of Head Impact and/or Brain Injury

**DOI:** 10.3389/fnhum.2022.933401

**Published:** 2022-07-25

**Authors:** Brent M. Roeder, Mitchell R. Riley, Xiwei She, Alexander S. Dakos, Brian S. Robinson, Bryan J. Moore, Daniel E. Couture, Adrian W. Laxton, Gautam Popli, Heidi M. Munger Clary, Maria Sam, Christi Heck, George Nune, Brian Lee, Charles Liu, Susan Shaw, Hui Gong, Vasilis Z. Marmarelis, Theodore W. Berger, Sam A. Deadwyler, Dong Song, Robert E. Hampson

**Affiliations:** ^1^Department of Physiology and Pharmacology, Wake Forest School of Medicine, Winston-Salem, NC, United States; ^2^Department Biomedical Engineering, Viterbi School of Engineering, University of Southern California, Los Angeles, CA, United States; ^3^Department of Neurosurgery, Wake Forest School of Medicine/Atrium Health Wake Forest Baptist, Winston-Salem, NC, United States; ^4^Department of Neurology, Wake Forest School of Medicine/Atrium Health Wake Forest Baptist, Winston-Salem, NC, United States; ^5^Department of Neurology, W. M. Keck School of Medicine, University of Southern California, Los Angeles, CA, United States; ^6^Department of Neurosurgery, W. M. Keck School of Medicine, University of Southern California, Los Angeles, CA, United States; ^7^Department of Neurology, Rancho Los Amigos National Rehabilitation Hospital, Los Angeles, CA, United States

**Keywords:** deep brain stimulation, hippocampus, memory, non-linear dynamics, traumatic brain injury, epilepsy, memory encoding, memory decoding

## Abstract

Rationale: Deep brain stimulation (DBS) of the hippocampus is proposed for enhancement of memory impaired by injury or disease. Many pre-clinical DBS paradigms can be addressed in epilepsy patients undergoing intracranial monitoring for seizure localization, since they already have electrodes implanted in brain areas of interest. Even though epilepsy is usually not a memory disorder targeted by DBS, the studies can nevertheless model other memory-impacting disorders, such as Traumatic Brain Injury (TBI). Methods: Human patients undergoing Phase II invasive monitoring for intractable epilepsy were implanted with depth electrodes capable of recording neurophysiological signals. Subjects performed a delayed-match-to-sample (DMS) memory task while hippocampal ensembles from CA1 and CA3 cell layers were recorded to estimate a multi-input, multi-output (MIMO) model of CA3-to-CA1 neural encoding and a memory decoding model (MDM) to decode memory information from CA3 and CA1 neuronal signals. After model estimation, subjects again performed the DMS task while either MIMO-based or MDM-based patterned stimulation was delivered to CA1 electrode sites during the encoding phase of the DMS trials. Each subject was sorted (*post hoc*) by prior experience of repeated and/or mild-to-moderate brain injury (RMBI), TBI, or no history (control) and scored for percentage successful delayed recognition (DR) recall on stimulated vs. non-stimulated DMS trials. The subject’s medical history was unknown to the experimenters until after individual subject memory retention results were scored. Results: When examined compared to control subjects, both TBI and RMBI subjects showed increased memory retention in response to both MIMO and MDM-based hippocampal stimulation. Furthermore, effects of stimulation were also greater in subjects who were evaluated as having pre-existing mild-to-moderate memory impairment. Conclusion: These results show that hippocampal stimulation for memory facilitation was more beneficial for subjects who had previously suffered a brain injury (other than epilepsy), compared to control (epilepsy) subjects who had not suffered a brain injury. This study demonstrates that the epilepsy/intracranial recording model can be extended to test the ability of DBS to restore memory function in subjects who previously suffered a brain injury other than epilepsy, and support further investigation into the beneficial effect of DBS in TBI patients.

## Introduction

If brain stimulation is to be of use to treat memory disorders, or develop a prosthetic for memory disorders, it is indispensable to address some of the limitations of the various approaches. We are aware that deep brain stimulation (DBS) can affect brain networks associated with memory, even if it is not clear precisely how that effect is produced (Reinhart and Nguyen, 2019). However, as cellular pathology progresses from injury or illness, those networks may not be intact or differ anatomically compared to patients without injury or illness. On the other hand, multi-site stimulation can restore memory function by “bypassing” damaged brain areas (Mohan et al., 2020), enhancing synaptic activity (Gondard et al., 2019), or promoting cellular repair and neurogenesis (Jones et al., 2020). Even in these cases, it is possible that a point can be reached where network-dependent stimulation is simply not effective. Thus, a true neuroprosthetic must function to replace lost cognitive function by not only bypassing, but replacing the output of hippocampus and associated memory regions (Berger and Glanzman, 2005; Deadwyler et al., 2017).

Our laboratories have demonstrated that a non-linear multi-input, multi-output model of hippocampal CA3–CA1 neuron interactions can be used to restore and even enhance hippocampal memory processing in rodents (Berger et al., 2011), non-human primates (Hampson et al., 2013), and even humans (Hampson et al., 2018b). This model extracts cell–cell interactions of the hippocampus (Berger et al., 2005), resulting in a prosthetic design that mimics the memory encoding function of the hippocampal CA3 and CA1 cell fields (Song et al., 2018). However, even this may be insufficient when both the encoding and recall functions of memory are already compromised before “normal” hippocampal neural activity can be recorded. Thus, a true memory prosthetic needs to be able to replace the patterned neural responses or “codes” associated with specific memory items regardless of brain state.

Prior research from these laboratories have demonstrated that the hippocampus (Hampson et al., 1999, 2004, 2005) and prefrontal cortex (Marmarelis et al., 2014; Opris et al., 2015) encode task-relevant information necessary for memory encoding and retrieval. Moreover, that information can be extracted and “transferred” between subjects in a limited manner (Deadwyler et al., 2013). Ongoing studies have focused on determining whether neural codes that represent memory instances can also be identified and facilitated with stimulation, by means of a MIMO-derived model (Roeder, 2021a,b). A memory decoding model (MDM) has been built for decoding memory information from hippocampal spiking data (She et al., 2021). The MDM provides signature functions representing the spatio-temporal characteristic of spike patterns most relevant to the memory. Such signature functions can be used to derive neural code-base stimulation patterns for each memory categories. Tests have demonstrated facilitation of memory encoding and recall with a duration of up to 75 min after stimulation, with preliminary indications of facilitated retention up to 24 h after stimulation (manuscripts in preparation).

With an understanding that the encoding of memory-relevant items by the hippocampus can be facilitated by a model of a neural prosthetic for human memory (Hampson et al., 2018b), we now turn our attention to neurological disorders and diseases that potentially impair memory to determine the utility of the neural prosthetic design for different medical cases. We show here the first demonstration of MIMO-derived model stimulation in subjects with normal intact memory, subjects with impaired memory but no history of head injury, and in subjects with a medical history of head impact of varying degrees.

## Materials and Methods

### Subjects

Twenty-five subjects were analyzed from the same pool of patients selected for the 2018 study (Hampson et al., 2018b). Subjects had medically-refractory focal epilepsy and were undergoing seizure monitoring and localization through the use of implanted intracranial depth electrodes, including surgical procedures, post-operative monitoring, and neurocognitive testing at one of the three sites participating in this study: Wake Forest Baptist Medical Center, Keck Hospital of USC, and Rancho Los Amigo National Rehabilitation Hospital. This study is part of the DARPA Restoring Active Memory (RAM) project. All procedures were reviewed and approved by each locations, Institutional Review Board (WFU IRB00023148, USC IRB#: HS-16-00068, RLANRH IRB#: 221) in accordance with the National Institutes of Health. Subjects provided voluntary written informed consent prior to participation separate from their consent for surgery.

### Memory Task

All memory testing utilized the two-part delayed-match-to-sample with delayed recognition (DMS-DR) assessment developed for the 2018 study (Hampson et al., 2018b). The DMS task consisted of 100–150 trials where each trial consisted of a single Sample phase image presented on a computer screen, a Sample response phase requiring a touch-screen response to the Sample image, a variable delay, a Match phase in which the Sample and 1–7 other images are displayed on-screen, and a Match response phase consisting of a touch-screen response to one of the images. Selection of the same image as the Sample was scored as a correct trial, while selection of any other image was scored as an error trial. DMS trials were separated by a 5 s intertrial interval.

The DR portion of testing commenced at minimum 15 min after completion of the DMS session. The total duration from start of DMS to completion of DR was 90 min or less. DR sessions always followed DMS stimulation sessions, but were not necessary when DMS results were recorded strictly for model generation. A DR trial consisted of presentation of three images at a time with a requirement that subjects rank the familiarity of each image. Each trial presented a Sample image from a prior DMS trial, a Non-match image (i.e., one of the other Match phase images) from the same DMS trial, and a Novel image that had not previously been seen by the subject. DR trials were presented in a randomized order compared to the sequence of DMS trials, and the locations of Sample, Non-match, and Novel images were also randomized to prevent the subject from memorizing sequence or position. Subjects ranked the familiarity of each image on a 0–5 scale, with 0 = not recognized, 1 = familiar escalating to 5 = certainty that the image had been seen in the prior DMS trials. Correct responses were scored as a trial in which the Sample image was ranked ≥3, and ≥the Non-matching image as well as the Novel image not being ranked at 5. Error trials were those trials in which Sample was ranked <3, Non-match images were ranked higher than the Sample, or if the Novel image was ranked=5.

Subjects were tested in two sessions; the first was 2 days after electrode implant and the second was 1 day before explant. These test days were selected because the patient would be on at least a partial dose of anti-seizure drugs and testing would not interfere with clinical seizure collection. The first test day collected data for model generation (see below) and consisted of a DMS session only. The second day consisted of 1 or 2 DMS-DR sessions. As only the second day utilized a DR session, we report only the analyses performed on these data sets.

### Hippocampal Neural Recording, Modeling and Stimulation

Neuronal recording procedure and submission of the recordings to USC for modeling were as reported previously (Hampson et al., 2018b). Two variations of the non-linear multi-input, multi-output MIMO model were generated: (1) A sparse dynamic model with continuous prediction of CA1 outputs from CA3 inputs (Song et al., 2009, 2018), and (2) a MDM decodes memory labels of images shown in DMS tasks based on both CA3 and CA1 neuronal spikes (She et al., 2021). Irrespective of the model used, DMS-DR testing remained the same, and results were not segregated into image categories or content except as follows: an upcoming publication (Roeder, 2021a) shows that one category (Building) was incorrectly designed (see Section “Memory Decoding and Stimulus Categories,” below), and the MDM stimulation always resulted in reduced performance on trials in which the category appeared. This category was omitted from analysis for the purpose of this report only.

Neuronal stimulation was controlled by a Matlab (Mathworks, Natick, MA, United States) script preprogrammed to emulate the MIMO and MDM models. Model-derived stimulation was applied to approximately two-third of all DMS trials, equally balanced between “positive” and “negative” stimulation trials. We specify that no stimulation was delivered during DR trials. All stimulation consisted of a 4 s multi-channel, spatio-temporally asynchronous biphasic square-wave pulse trains with a maximum continuous frequency of 20 Hz (Hampson et al., 2018b) commencing with Sample presentation. Positive stimulation-trains were derived from either the continuous MIMO model or the discrete MDM model corresponding to the category of the Sample image. Negative stimulation-trains consisted of either a random spatial–temporal pattern (counterbalancing the MIMO model), or were derived from the discrete MDM model for a different category from the Sample image. The remaining one-third of DMS trials received no stimulation.

### Memory Decoding and Stimulus Categories

In human memory decoding, we used image labels to represent visual memory information. Such image labels were obtained from normal volunteers giving scores to DMS stimulus images through an online survey system. Five main categories (i.e., Animal, Building, Plant, Tool, and Vehicle) were selected as the main decoding target categories. The MDM (She et al., 2021) takes hippocampal CA3 and CA1 spiking activities as model inputs and labels of the five image categories as output. It has been proved to be able to identify spatio-temporal characteristics of spike patterns most relevant to the memory categories from ensemble spike patterns. Furthermore, model-based stimulation patterns can be derived based on MDM coefficients to elicit specific memories. In addition, parallel computing strategies were utilized to accelerate model estimation to ensure such model-based stimulation patterns can be calculated on time (She et al., 2022).

Note that survey respondents selected the features that best fit the image, but were not necessarily feature descriptions normally be associated with an image. Thus, there were some deviations from strict categories – Building category included houses, churches, office buildings, arenas, bridges, and architectural features. The Tool category is made up of items best described as “arts and crafts consumables.” In the former case, the category was quite broad, and less defined, and in the latter case, the category was narrow and quite coherent.

Images corresponding to a given category were used for the Sample and Match image within DMS trials, while images from other categories were used for Non-match images. The actual categories used did not affect the MIMO model tests, nor were categories treated separately for the MDM results reported here. A preliminary analysis of MDM stimulation revealed inconsistency with respect to effects of the Building category (Roeder, 2021a), suggesting that the “Building” category may not necessarily be a coherent category or it may be one for which “building” is not a good descriptor—merely the closest available from the survey. For that reason, “Building” trials were omitted from this analysis; the other four categories were combined into a single indication for comparison with similar results from MIMO stimulation.

### Brain Injury and Memory Impairment

Since all subjects in this study had a history of epilepsy, it was expected that nearly all would show some form of memory impairment or brain injury. Medical records for all subjects were examined to determine whether a prior history of brain injury was reported, or whether pre-surgical neuropsychological assessment revealed memory impairment. For the purposes of this study, a classification of traumatic brain injury (TBI) was reserved for subjects whose epilepsy diagnosis commenced with a report of a serious fall, a severe head impact or motor vehicle accident. A classification of RMBI was applied to subjects with a history of head-impact through sports, falls or whose MRI showed evidence of prior impact trauma. Control subjects were those whose medical history ruled out TBI or RMBI. Furthermore, subjects were rated as Normal Memory if pre-surgical neuropsychological assessment observed no performance deficit in standardized memory assessments. Subjects with a diagnosis of mild-to-moderate memory, irrespective of spatial, verbal, or lateralization, were rated as Impaired Memory. No subjects in this study received a diagnosis of greater than moderate memory impairment.

## Results

A total of 25 Phase II (intracranial monitoring) epilepsy patients were tested as subjects in this study (Table 1). A total of 24 subjects were tested with either MIMO or MDM stimulation computed from a recording day at least 3 days prior to the stimulation day. Of those subjects, nine subjects were tested exclusively with stimulation derived from the MIMO model, eleven were tested exclusively with stimulation derived from MDM, and four were tested (separately) with stimulation derived from both models.

**TABLE 1:** Patient demographics.

Patient	Test site	TBI type	Memory	Sex	Age	MIMO	MDM
KECK06	KHUSC	TBI	Normal	M	42	√	
KECK08	KHUSC	Control	Impaired	M	26	√	√
KECK15	KHUSC	Control	Impaired	F	20		√
RANCHO01	RLANRH	RMBI	Impaired	M	35		√
RANCHO07	RLANRH	Control	Normal	M	35		√
WAKE14	WFSM	RMBI	Impaired	M	35	√	
WAKE15	WFSM	TBI	Impaired	M	45	√	
WAKE16	WFSM	Control	Normal	M	21	√	
WAKE17	WFSM	TBI	Impaired	F	31	√	
WAKE18	WFSM	Control	Normal	F	55	√	
WAKE19	WFSM	TBI	Normal	F	33	√	
WAKE20	WFSM	Control	Normal	F	31	√	√
WAKE21	WFSM	TBI	Impaired	F	26	√	√
WAKE22	WFSM	RMBI	Impaired	M	48		√
WAKE23	WFSM	RMBI	Normal	F	51		√
WAKE24	WFSM	Control	Normal	F	33		√
WAKE25	WFSM	Control	Impaired	F	67		√
WAKE26	WFSM	Control	Impaired	M	23		
WAKE28	WFSM	RMBI	Impaired	F	55		√
WAKE29	WFSM	Control	Normal	F	38		√
WAKE30	WFSM	RMBI	Normal	M	55		√
WAKE34	WFSM	TBI	Normal	F	40		√
WAKE35	WFSM	RMBI	Impaired	M	20	√	
WAKE36	WFSM	TBI	Normal	M	42	√	
WAKE37	WFSM	Control	Impaired	F	41	√	√

*Subjects tested in the report. TEST SITE: KHUSC = Keck Hospital/School of Medicine, University of Southern California; RLANRH = Rancho Los Amigos National Rehabilitation Hospital; WFSM – Wake Forest School of Medicine (Atrium Health Wake Forest Baptist). TBI TYPE – TBI = Traumatic Brain Injury, subject has history of serious head injury (may include loss of consciousness); RMBI = Repeated Mild-Moderate Brain Injury, subject history indicates falls, sports injuries, or head impacts with no loss of consciousness; Control = no history of head impact. MEMORY - Normal = no evaluation of memory impairment in pre-surgical neuropsychological evaluation; Impaired = pre-surgical neuropsychological evaluation included an assessment of mild-to-moderate memory impairment. MIMO = subject was tested with hippocampal stimulation derived from non-linear multi-input, multi-output (MIMO) model of hippocampal CA1 neural ensemble activity. MDM = subject was tested with hippocampal stimulation derived from non-linear Memory Decoding Model (MDM) of hippocampal CA1 neural ensemble activity.*

**FIGURE 1:**
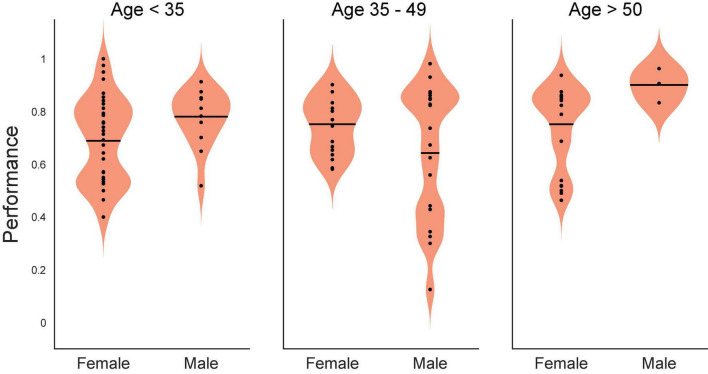
Statistical interaction plot for the ANOVA. An ANOVA (SAS GLM, SAS Institute, Cary, NC, United States) of control (Non-stimulated) delayed-match-to-sample with delayed recognition (DMS-DR) test performance per subject sorted according to age (<35, 35–49, >50 years of age) and sex (Male, Female). DMS-DR performance was the dependent variable, subject was a continuous variable, while sex and age were independent discrete classification variables. The statistical interaction plot for the two-way ANOVA is shown.

As a preliminary statistical screen, an ANOVA (SAS GLM, SAS Institute, Cary, NC, United States) of control DMS performance was performed to assess whether there were baseline differences with respect to age or sex (as provided in Table 1). Since subject sex was a binary classification, subject age was converted to a classification variable as well by grouping subjects into ages <35 years of age, 35–49 and ≥50 years of age. While not a perfectly uniform distribution, this grouping yielded 12 females: 5 @ < 35, 3 @ 35–49 and 4 @ ≥ 50; and 13 males: 4 @ < 35, 7 @ 35–49 and 2 @ ≥ 50. The ANOVA yielded a significant effect of the model [*F*(5,63) = 3.22, *p* < 0.01], and the main effect of age [*F*(2,63) = 5, *p* < 0.01], with non-significant main effects of sex [*F*(1,63) = 0.59, *p* > 0.4] and non-significant interaction [*F*(2,63) = 2.75, *p* > 0.05] term. The graph of the interaction plot shows considerable overlap in the main subject groups (Figure 1). Despite some non-significant differences in DMS-DR performance by age, the overlap in distribution of scores at all ages allowed us to proceed with analysis of TBI and memory impairment without an age subset.

During stimulation testing sessions, DMS trials received: (1) Positive stimulation, consisting of model-derived stimulation patterns that matched either the MIMO model prediction of CA1 firing from continuous CA3 input or MDM prediction of CA1 firing from CA3 input for a given trial type (image category); (2) Negative stimulation, consisting of either random patterns to mimic a non-specific MIMO CA1 spatio-temporal firing or MDM stimulation from a different trial type; or (3) No stimulation (NoStim). The three stimulation conditions were balanced within a session to provide one third of DMS trials meeting each stimulation condition. The Positive stimulation patterns were modeled to produce CA1 ensemble firing with the highest probability of correlation with correct DMS-DR performance. Negative stimulation patterns were intended to counter-balance the Positive stimulation but providing a spatio-temporal pattern that was either randomized or not correlated with the trial type. NoStim trials captured the normal range of subject performance without the influence of the hippocampal stimulation. All subjects performed at least 100 DMS-DR trials, with some subjects performing as many as 150 trials in a single ninety-minute test session. Subjects that were tested with more than one model were tested on the same day with at least one hour between test sessions.

### Facilitation as a Function of Brain Injury Status

Mean (±SEM) DMS-DR performance sorted by TBI status is shown in Table 2. Due to the low “n” in several categories, a “Combined” model performance group (top rows in Table 2) shows DMS-DR performance for model-stimulated sessions irrespective of whether the model was MIMO or MDM-based. For the four subjects who received both MIMO and MDM-based stimulation, the performance for Positive, Negative, and NoStim trials was averaged across models. Note that for purposes of comparison between models, the MDM model results are averaged across performance of four trial image-type categories (see Section “Materials and Methods”). Instances in which only one subject’s data was available do not report SEM.

**TABLE 2:** Delayed-match-to-sample – delayed recognition (DMS-DR) performance by TBI status.

	#Subjects	Positive stim	NoStim	Negative stim	%Change positive stim	%Change negative stim
**ALL STIM**						
Control	11	81.1% ± 3.8%	70.6% ± 4.1%	71.9% ± 5.4%	14.8%	1.8%
RMBI	7	75.4% ± 7.2%	65.1% ± 8.3%	61.8% ± 11.6%	15.8%	–5.0%
TBI	7	80.5% ± 3.1%	70.6% ± 7.9%	60.2% ± 7.8%	14.1%	–14.8%
**MDM STIM**						
Control	10	79.9% ± 3.7%	70.2% ± 4.9%	71.7% ± 5.5%	13.8%	2.2%
RMBI	5	79.1% ± 9.3%	70.2% ± 9.5%	71.7% ± 9.4%	12.7%	2.2%
TBI	4	78.6% ± 3.2%	61.8% ± 9.7%	58.5% ± 7.6%	27.3%	–5.4%
**MIMO STIM**						
CONTROL	4	89.7% ± 3.9%	66.0% ± 6.5%	67.5% ± 8.5%	36.0%	2.4%
RMBI	2	65.9% ± 10.0%	52.3% ± 17.9%	12.5% ± .0%	26.1%	–76.1%
TBI	5	80.8% ± 4.1%	73.6% ± 9.2%	59.8% ± 11.0%	9.8%	–18.8%

*Stimulated and non-stimulated task performance by TBI Status for subjects receiving CA1 stimulation based on MDM and MIMO models. Positive stim = stimulation patterns derived from modeling correct DMS trials (MIMO) or from MDM patterns that were congruent with the content classification of the Sample/Match image. NoStim = trials with no stimulation delivered. Negative stim = trials with random stimulation patterns (MIMO) or from MDM that were not specific to the content classification of the Sample/Match image). Stimulation was only delivered during the Sample phase of DMS trials, and not delivered during Match phase, nor during the DR assessment. %Change = percentage of increase (+, no symbol) or decrease (–) compared to NoStim.*

The two columns at the right compare Positive stimulation trials to NoStim trials, and Negative stimulation trials to NoStim trials, respectively. The increase (positive values) or decrease (negative value) compared to absence of stimulation indicates the effectiveness of Model-based stimulation to alter memory retention in the DMS-DR task. From these results, the MIMO and MDM models had varying degrees of effectiveness, but the combined results show effective facilitation of memory retention across control, RMBI, and TBI conditions. Effects of MIMO stimulation on Control subjects are consistent with the prior peer-reviewed report (Hampson et al., 2018b); while reduced from MIMO, the effectiveness of MDM-based stimulation is consistent with non-peer-reviewed results reported by this laboratory (Hampson et al., 2018a,^2019^). While RMBI results were similar to Control for MDM and MIMO, TBI subjects appeared to obtain greater facilitation from MDM-based than MIMO-based stimulation.

### Facilitation as a Function of Pre-existing Memory Status

Our prior study (Hampson et al., 2018b) suggested that baseline memory status did not significantly alter the effectiveness of memory facilitation by MIMO model-based stimulation. Since the earlier study utilized fewer subjects, and the influence of pre-existing memory status was not evaluated with either MDM-based stimulation, or with TBI status, subject results were sorted according to baseline memory function as reported by the pre-surgical neuropsychological assessment. Table 3 shows that baseline memory performance does in fact appear to influence effects of both MIMO and MDM-based stimulation, with the MIMO model producing almost twice the facilitation of memory in subjects who already exhibit memory impairment. While the differential between impaired and normal subjects is less for MDM-based stimulation, it is nonetheless increased, and this differential carries through to the combined performance across models. It is worth noting the magnitude of the differential between Positive MIMO stimulation and Negative (randomized) stimulation for impaired subjects. Statistical analysis via ANOVA testing main effects of TBI status (RMBI, TBI, Control), memory status (Impaired, Normal) and the interaction of TBI and memory yielded a highly significant overall effect [*F*(5,180) = 10.70, *p* < 0.001], the main effect of memory status [*F*(1,180) = 24.11, *p* < 0.001] and the interaction of TBI*memory [*F*(2,180) = 13.57, *p* < 0.001], but non-significant main effects of TBI [*F*(2,180) = 1.12, *p* > 0.3]. These results confirm the trends listed in Tables 2, 3.

**TABLE 3:** Delayed-match-to-sample – delayed recognition performance by baseline memory function.

	#Subjects	Positive stim	NoStim	Negative stim	%Change positive stim	%Change negative stim
**ALL STIM**						
Impaired	12	75.5% ± 3.9%	61.9% ± 5.1%	56.4% ± 6.4%	22.0%	–8.8%
Normal	12	83.0% ± 3.5%	76.1% ± 4.3%	75.9% ± 5.2%	9.1%	–0.3%
**MDM STIM**						
Impaired	9	77.8% ± 4.6%	65.2% ± 5.9%	63.7% ± 4.9%	19.3%	–2.4%
Normal	10	80.9% ± 4.1%	71.4% ± 5.5%	73.9% ± 6.4%	13.4%	3.6%
**MIMO STIM**						
Impaired	5	73.1% ± 5.2%	54.9% ± 6.8%	40.3% ± 10.9%	33.2%	–26.6%
Normal	6	88.2% ± 3.2%	77.1% ± 6.4%	73.3% ± 6.6%	14.5%	–4.9%

*Stimulated and non-stimulated task performance by baseline memory function for subjects receiving CA1 stimulation based on MDM and MIMO models. Positive stim = stimulation patterns derived from modeling correct DMS trials (MIMO) or from MDM patterns that were congruent with the content classification of the Sample/Match image. NoStim = trials with no stimulation delivered. Negative stim = trials with random stimulation patterns (MIMO) or from MDM that were not specific to the content classification of the Sample/Match image). Stimulation was only delivered during the Sample phase of DMS trials, and not delivered during Match phase, nor during the DR assessment. %Change = percentage of increase (+, no symbol) or decrease (–) compared to NoStim.*

### Combined Brain Injury Plus Memory Status

Based on the indications of variable effectiveness due to pre-existing memory function, the subjects were further sorted according to TBI status and memory function. The results of the sorting are shown in Table 4. There were no RMBI+Normal subjects tested with MIMO-based stimulation; therefore that row was omitted from Table 4. There were also several conditions under which only one subject was tested. Those values are included in the table, but any interpretation of those results is premature. Highlighted cells in the columns for percentage change in DMS-DR performance due to model-based stimulation indicate conditions with at least two subjects, and at least 2.25 × SEM differences (approx. *p* < 0.01) from NoStim.

**TABLE 4:** Delayed-match-to-sample – delayed recognition performance by TBI and memory.

		#Subjects	Positive stim	NoStim	Negative stim	%Change positive stim	%Change negative stim
**ALL STIM**							
Control	Impaired	4	83.9% ± 1.6%	73.6% ± 5.0%	75.3% ± 5.5%	13.9%**	2.2%
	Normal	6	79.2% ± 6.3%	68.6% ± 6.1%	69.2% ± 8.9%	15.4%	0.9%
RMBI	Impaired	5	69.0% ± 8.5%	58.6% ± 10.5%	46.7% ± 11.3%	17.6%	–20.4%
	Normal	2	91.3% ± 5.0%	81.1% ± 2.2%	92.2% ± 1.6%	12.5%*	13.6%**
TBI	Impaired	3	75.1% ± 3.9%	51.5% ± 3.8%	44.2% ± 7.1%	45.8%**	–14.2%
	Normal	4	84.6% ± 3.7%	84.9% ± 7.3%	76.1% ± 6.2%	–0.3%	–10.4%
**MDM STIM**							
Control	Impaired	4	83.1% ± 2.3%	74.6% ± 4.3%	74.0% ± 6.4%	11.5%*	–0.7%
	Normal	6	77.8% ± 6.0%	67.3% ± 7.8%	69.9% ± 8.9%	15.6%	3.9%
RMBI	Impaired	3	71.0% ± 14.2%	62.9% ± 15.3%	58.0% ± 7.8%	13.0%	–7.7%
	Normal	2	91.3% ± 5.0%	81.1% ± 2.2%	92.2% ± 1.6%	12.5%*	13.6%**
TBI	Impaired	2	77.2% ± 1.4%	49.9% ± 3.4%	51.3% ± 1.3%	54.6%**	2.8%
	Normal	2	80.1% ± 7.4%	73.7% ± 16.5%	65.6% ± 15.6%	8.7%	–10.9%
**MIMO STIM**							
Control	Impaired	1	83.3%	58.1%	68.6%	43.3%	18.0%
	Normal	3	91.8% ± 4.6%	68.6% ± 8.5%	67.0% ± 13.9%	33.9%**	–2.3%
RMBI	Impaired	2	65.9% ± 10.0%	52.3% ± 17.9%		26.1%	–76.1%
	Normal	0					
TBI	Impaired	2	75.1% ± 7.8%	55.8% ± 11.5%	40.0% ± 10.0%	34.7%*	–28.3%
	Normal	3	84.7% ± 4.3%	85.5% ± 7.7%	79.5% ± 4.5%	–1.0%	–7.0%

Stimulated and non-stimulated task performance sorted by TBI and memory for subjects receiving CA1 stimulation based on MDM and MIMO models. Positive stim = stimulation patterns derived from modeling correct DMS trials (MIMO) or from MDM patterns that were congruent with the content classification of the Sample/Match image. NoStim = trials with no stimulation delivered. Negative stim = trials with random stimulation patterns (MIMO) or from MDM that were not specific to the content classification of the Sample/Match image). Stimulation was only delivered during the Sample phase of DMS trials, and not delivered during Match phase, nor during the DR assessment. %Change = percentage of increase (+, no symbol) or decrease (–) compared to NoStim. Conditions with significant increase or decrease in DR performance relative to NoStim (*p < 0.01, **p < 0.001 by pairwise linear contrasts) are indicated by asterisks. Note, there were no Normal Memory RMBI subjects tested with MIMO stimulation.

**FIGURE 2:**
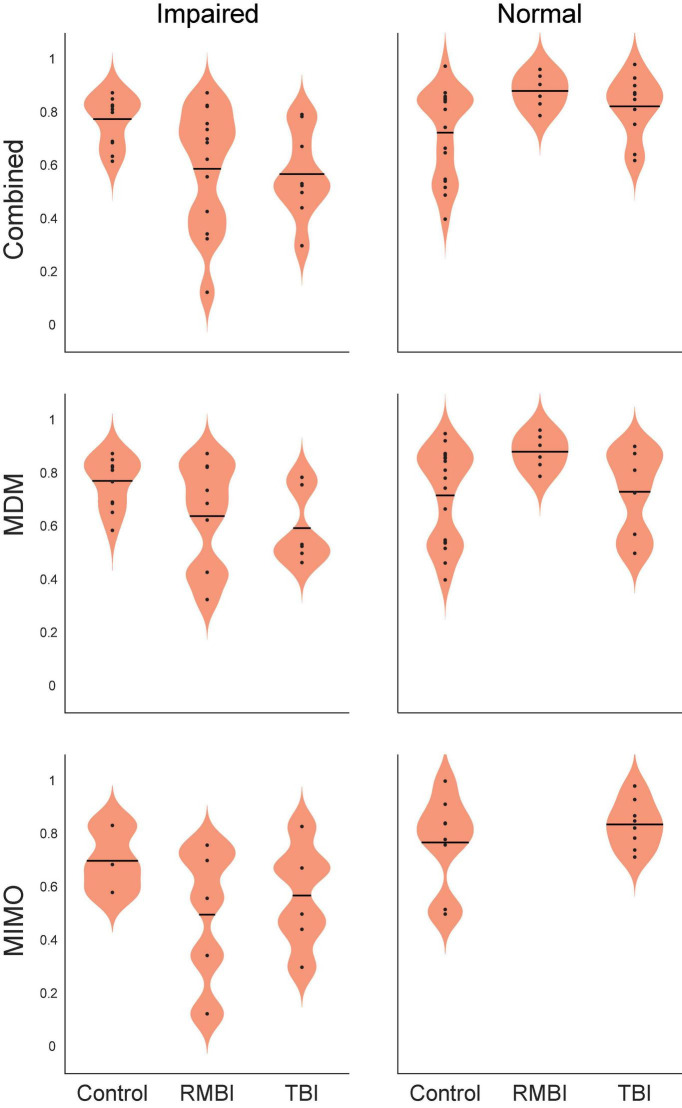
Statistical interaction plot for the ANOVA by model. Performance by interaction traumatic brain injury (TBI)*memory plots for ANOVAs performed for each of the stimulation models. DMS-DR performance per subject (dependent variable) was modeled with independent variables of TBI-type [(Control, TBI, repeated and/or mild-to-moderate brain injury (RMBI)] and memory status (Normal, Impaired).

In support of the stim effects in Table 4, we can confirm significant effects of memory and the interactions between TBI status and memory for individual stimulation models for the *Combined* task performance: main effect of memory [*F*(2,63) = 11.24, *p* < 0.001] and interaction TBI*memory [*F*(1,63) = 8.38, *p* < 0.001]; and for *MDM* task performance: main effect of memory [*F*(1,50) = 5.89, *p* < 0.01], and interaction TBI*memory [*F*(2,50) = 4.88, *p* < 0.01]. Interestingly, for the MIMO model, there was no significant interaction, but significant main effects of TBI [*F*(2,25) = 3.88, *p* < 0.05] and memory [*F*(1,25) = 6.59, *p* < 0.01]. The interaction plots in Figure 2 depict the performance changes associated with each of these analyses.

Comparisons highlighted in Table 4, right (%Change) are supported by ANOVA and linear contrasts of the derived measures of percent change from NoStim for positive and negative stim. The overall ANOVA was significant [*F*(5,59) = 3.44, *p* < 0.01]. Again, there was not a significant main effect of TBI [*F*(2,59) = 0.42, *p* > 0.6] but there were significant main effects of memory [*F*(1,59) = 4.9, *p* < 0.05] and interaction TBI*memory [*F*(2,59) = 5.74, *p* < 0.01]. Orthogonal pairwise contrasts were computed using this model. Asterisks in Table 4 indicate conditions in which positive or negative stim results differed from NoStim with *p* < 0.001.

### Summary of Stimulation-Induced Changes in Memory

To compute statistical comparisons of the effects of stimulation, normalized difference scores were calculated by subtracting the mean Control-NoStim DMS-DR performance and dividing by within-subject standard deviation to yield standardized scores with mean = 0 and SD = 1. Two additional derived factors – (1) the difference between NoStim performance for a given subject and the overall mean of NoStim Control trial DMS-DR performance, and (2) Delta – the difference between No-Stim trial DMS-DR performance and the Positive (or Negative) stim trial DMS-DR performance for a given subject.

The overall multi-factor ANOVA on effects of the stim model, TBI status, memory status and stimulation type yields a significant effect of the model, where [*F*(50,104) = 2.59, *p* < 0.001]. Main effects analyses for individual factors and interaction yielded Model type [*F*(2,104) = 0.36, *p* = 0.70]; TBI status [*F*(2,104) = 2.58, *p* = 0.08]; Memory status [*F*(1,104) = 22.85, *p* < 0.001]; and Stim type [*F*(2,104) = 21.87, *p* < 0.001]. The only significant interaction term (of all 2-way, 3-way, and 4-way interactions) was TBI status × Memory status [*F*(2, 104) = 16.46, *p* < 0.001].

**FIGURE 3:**
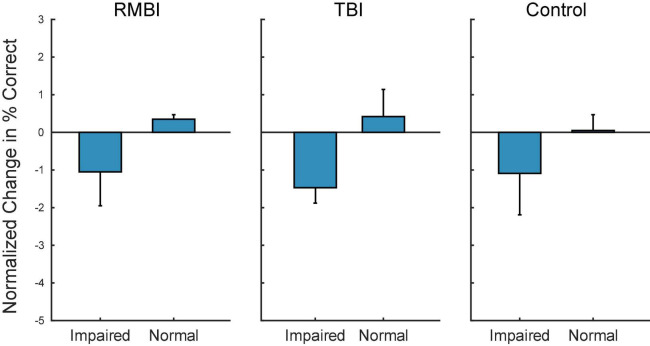
Subject–Condition differences in non-stimulated DMS-DR performance. Individual subject DMS-DR results were normalized by subtracting overall non-stimulated trial performance from control performance (i.e., non-stim DMS-DR from Control/Normal subjects), and dividing by individual subject standard deviation. The resulting differences in performance for the RMBI/TBI by Impaired/Normal memory status is plotted as mean (±intersubject SEM) normalized difference from baseline, control performance in the absence of stimulation. As expected, memory impaired subject performance the DMS-DR task worse than non-impaired subjects. (Note, since control performance was aggregated from all Control/Normal subjects, the bar and SEM for Control/Normal indicate individual subject variability.)

Figure 3 shows the summary bargraph of Normalized change in DMS-DR Percent Correct for NoStim trials sorted by TBI status and Memory status. The normalization baseline was composed of non-stim trials specifically gathered from the Control/Normal subjects, supplying mean and standard deviation for computation of normalized values (e.g. standard scores). Performance for all memory-Impaired subjects (irrespective of stimulation model) was below the control mean for NoStim trials. Moreover, performance for Normal memory subjects was slightly elevated for the TBI and RMBI groups. Compared to baseline non-stimulated DMS-DR performance, memory-impaired subjects performed *all* DMS-DR trials at a performance level below that of normal memory subjects.

**FIGURE 4:**
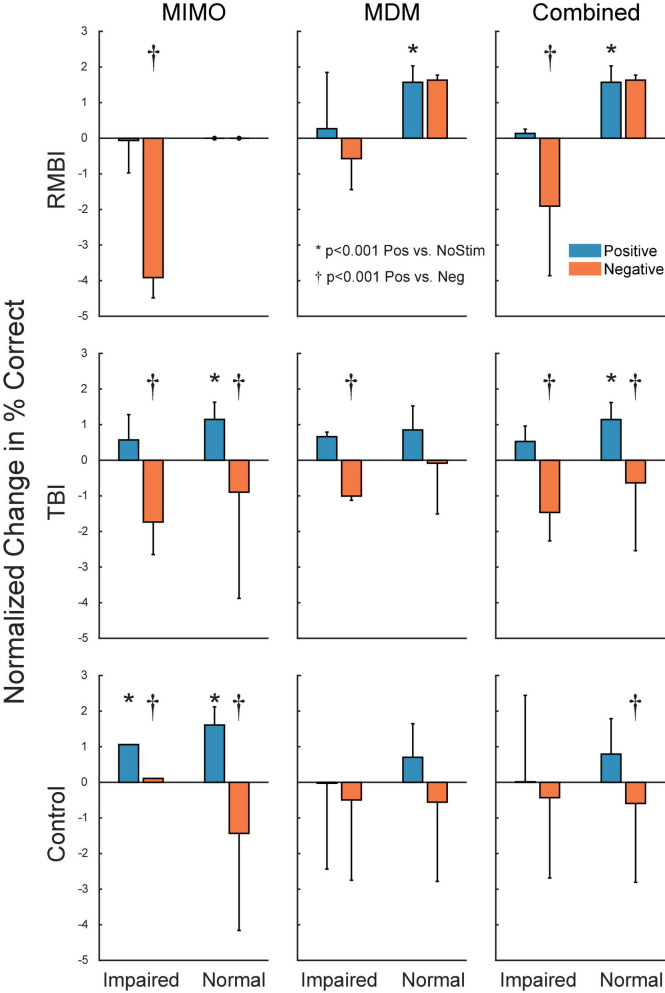
Subject–Condition differences in stimulated DMS-DR performance. Individual subject DMS-DR results were normalized by subtracting mean within-subject NoStim positive and negative pattern stimulated trial performance, and dividing by individual subject standard deviation to produce standard scores. Scores were then sorted by TBI status, stimulation model and presence or absence of memory impairment, and plotted as normalized mean (±inter-subject SEM) differences in DMS-DR performance. Individual linear contrasts were computed using paired-differences and mean standard error (MSE) from the overall multi-factor ANOVA. Asterisks (*) indicate conditions with significant differences between positive stim and NoStim conditions. Daggers (†) indicate statistically significant differences between positive and negative stim conditions.

Figure 4 shows the summary of interactions between TBI and Memory status for each of the stimulation models. To identify individual effects of Positive stimulation of DMS-DR performance, orthogonal pairwise linear contrasts (Neter and Wasserman, 1974) were computed between Positive and NoStim for each condition. Asterisks (*) in Figure 4 indicate those conditions under which there is a significant difference between positive stimulation and the normalized NoStim DMS-DR performance. To identify differential effects of stimulation, orthogonal pairwise linear contrasts were computed between Positive and Negative stimulation for each condition. Daggers (†) indicate significant differences between Positive and Negative stim conditions, indicating modulation of memory via stimulation even when that positive stim does not significantly increase DMS-DR performance. All comparisons are [*F*(1,104) > 15.09, *p* < 0.001] via orthogonal linear contrasts adjusted for multiple comparisons. (Neter and Wasserman, 1974)

## Discussion

The question of whether human memory can be modulated via intracranial stimulation of hippocampus (or entorhinal cortex) is one that suffers slightly from comparison with DBS fixed stimulation techniques (Mohan et al., 2020). Jacobs et al. (Jacobs et al., 2016; Goyal et al., 2018) stimulated hippocampus with high, fixed-frequency stimulation and noted impairment in working and episodic memory. Other studies by Aghajan et al. (2017), Titiz et al. (2017), Mankin et al. (2021) have stressed that the stimulation pulse-train frequency is essential to whether or not hippocampal/entorhinal stimulation is effective or not.

Theta-like activity in the 3.5–7 Hz band has been shown to be an important contributor to hippocampal-dependent memory processing (Kota et al., 2020; Kragel et al., 2020; Nicolas et al., 2021). Theta-burst stimulation of hippocampus improves memory (Titiz et al., 2017; Tambini et al., 2018; Jun et al., 2020), possibly by synchronizing theta power (Karakas, 2020), theta-gamma coupling (Jones et al., 2020), or via synaptic plasticity based on theta frequency activation of neuronal circuits in the temporal lobe (Tsanov and Manahan-Vaughan, 2009; Larson and Munkácsy, 2015).

Given that DBS-like high-frequency stimulation is most effective in facilitating memory when applied outside hippocampus (Ezzyat et al., 2018; Kucewicz et al., 2018), but not when applied to hippocampus (Jacobs et al., 2016), it is therefore possible that the MIMO model-based hippocampal stimulation applied here and previously (Hampson et al., 2018b; Roeder, 2021a) is effective at facilitating human short-term memory precisely because the stimulation frequency is capped at ≤20 Hz. Moreover, the stimulation is also based on a closed-loop approach that models the stimulation pattern on existing neural ensemble firing patterns associated with successful memory function (Song et al., 2016, 2018; She et al., 2021). Overall, the results presented here indicate that both MIMO or MDM-based stimulation can be effective in facilitating memory retention (up to 75 min) across all subjects irrespective of TBI status or pre-existing memory function. Irrespective of baseline memory function (impaired vs. normal), the MIMO model produces at least double the facilitation compared to the MDM model (Table 4). In all likelihood, this is due to the nature of the MDM model which is segmented into individually-specific discrete codes according to the categorization of the Sample image presented in DMS trials. It is possible, that the smaller effect of MDM-based stimulation is due to variability between categories within a subject, rather than variability between subjects.

The 2018 report from this laboratory did not reveal a significant effect of MIMO-based stimulation on subjects with impaired memory function (Hampson et al., 2018b), but concluded that at minimum, MIMO-model based stimulation was at least as effective across categories of pre-existing memory function. The present study includes more than three times as many subjects, and Table 3 shows that the MIMO was at least twice as effective in memory-impaired subjects, while the MDM-based stimulation was at least one standard error (SEM) more effective in impaired subjects compared to subjects with normal memory function.

The collation of subjects by TBI status and memory function in Table 4 shows that across subjects, the combined stimulation models were most effective in Impaired Controls, *Normal* RMBI and Impaired TBI subjects. Normal RMBI subjects also benefitted from Negative or non-category-specific MDM stimulation. This improvement overshadowed the decline in performance from the memory-impaired RMBI subjects and led to an overall increase with Negative MDM stimulation. This was the only case in which stimulation not specific to the model was facilitatory, and of course it raises the question of whether simply any low-frequency stimulation could be facilitatory in these subjects. However, it is quite subject specific (i.e. only Normal-memory, RMBI subjects). We are aware that there are some issues with respect to the composition of the categories used to generate that model (see Section “Materials and Methods”) and it is possible that these results indicate cross-category similarities. On the other hand, Mankin and Fried (2020) suggest that a key component of the MIMO success derives from underlying low frequency and sparsity of the stimulation (Mankin and Fried, 2020). As mentioned above, theta-band stimulation has been significantly involved with hippocampal memory processing and our MDM stimulation has a significant theta-component. Therefore, we theorize that even non-specific stimulation likely creates an improvement if a patient does not have impaired memory. This is a subject of ongoing investigation.

Figure 3 demonstrates that, as expected, subjects with a neuropsych evaluation that included mild-to-moderate memory impairment scored lower on DMS-DR task performance. Normalizing the data to evaluate effects of stimulation irrespective of baseline DMS-DR performance (Figure 4) shows that the MIMO model significantly improved DMS-DR performance in TBI and Control (No-TBI) subjects, while the MDM stimulation was most effective in RMBI subjects (asterisks, Figure 4). The MIMO model was most effective in all RMBI, TBI and Control subjects, irrespective of memory impairment. In TBI subjects, both models were quite effective, particularly in memory-impaired subjects (daggers, Figure 4). One possibility why MIMO stimulation produces more of a differential in memory performance is because MIMO negative stimulation consisted of randomized stim patterns, while for MDM the negative stim was not random, but a pattern associated with a different category, which might have some cross-over benefit (see top graph in Figure 4, MDM-Normal) (Roeder, 2021a). Future studies will explicitly examine difference in possible partial benefit of out-of-category stimulation vs. randomized stimulation patterns.

### Summary

These results suggest that controls and subjects with a diagnosis of TBI receive equal benefit from memory-facilitating stimulation. Biomimetic MIMO based stimulation is more effective than MDM based stimulation. Model-based stimulation is more effective in subjects with a prior medical history of memory impairment, leading to maximal benefit in TBI subjects with memory impairment.

Effects of stimulation designed to emulate a neural prosthetic are different in subjects with a diagnosis of Repeated Mild-to-Moderate Brain Injury (RMBI, i.e., falls, concussions, sports injuries). Non-memory-impaired RMBI subjects received the most benefit from the model-based stimulation, while impaired RMBI subjects received benefit from the non-specific stimulation from the MDM.

These results suggest that both models have the potential to improve memory function in patients with neurological impairments caused by disease or injury.

## Data Availability Statement

The raw data supporting the conclusions of this article will be made available by the authors, without undue reservation.

## Ethics Statement

The studies involving human participants were reviewed and approved by Wake Forest School of Medicine IRB (IRB00023148), Keck Hospital of the University of Southern California IRB (S-16-00068), and Rancho Los Amigos National rehabilitation Hospital IRB (IRB#221). The patients/participants provided their written informed consent to participate in this study.

## Author Contributions

RH was project PI and supervised all aspects of design, data collection, data analysis, and manuscript preparation. DS was co-PI of the overall clinical project, and shares senior authorship with RH. BMR, MR, and AD participated in study design, experimental control software, data collection (at WFSM), analysis, and manuscript preparation. BMR, XS, BM, and DS participated in data collection at KHUSC and RLANRH. XS, BR, BM, VM, TB, and DS participated in MIMO and MDM model development. DC, AL, BL, and CL provided IRB and study design, performed neurosurgical procedures at their respective institutions. GP, HMC, MS, CH, GN, SS, and HG provided IRB & study design, diagnostic, patient monitoring, supervised data collection, and neurological care for the subjects at their respective institutions. VM, TB, DS, SD, and RH participated in project management and study design. TB and DS were subcontract PIs for USC; SD was originating PI (now emeritus) for the study. Portions of this work appeared in BR’s Ph.D. dissertation. All authors agree to be accountable for the content of this work.

## Conflict of Interest

RH discloses a current consulting and advisory relationship with Braingrade, Inc., a component of Engram (Holding), Inc., a Delaware C-Corporation. This relationship was not in effect at the time of the study. The remaining authors declare that the research was otherwise conducted in the absence of any other commercial or financial relationships that could be construed as a potential conflict of interest.

## Publisher’s Note

All claims expressed in this article are solely those of the authors and do not necessarily represent those of their affiliated organizations, or those of the publisher, the editors and the reviewers. Any product that may be evaluated in this article, or claim that may be made by its manufacturer, is not guaranteed or endorsed by the publisher.

## References

[B1] Aghajan Z. M. Schuette P. Fields T. A. Tran M. E. Siddiqui S. M. Hasulak N. R. (2017). Theta Oscillations in the Human Medial Temporal Lobe during Real-World Ambulatory Movement. *Curr. Biol.* 27 3743–3751.e3. 29199073 10.1016/j.cub.2017.10.062 PMC5937848

[B2] Berger T. W. Ahuja A. Courellis S. H. Deadwyler S. A. Erinjippurath G. Gerhardt G. A. (2005). Restoring lost cognitive function. *IEEE Eng. Med. Biol. Mag.* 24 30–44. 10.1109/memb.2005.1511498 16248115

[B3] Berger T. W. Glanzman D. L. (2005). *Toward Replacement Parts for the Brain.* Cambridge, MA: MIT Press.

[B4] Berger T. W. Hampson R. E. Song D. Goonawardena A. Marmarelis V. Z. Deadwyler S. A. (2011). A cortical neural prosthesis for restoring and enhancing memory. *J. Neural Eng.* 8:046017. 10.1088/1741-2560/8/4/046017 PMC3141091 21677369

[B5] Deadwyler S. A. Berger T. W. Sweatt A. J. Song D. Chan R. H. Opris I. (2013). Donor/recipient enhancement of memory in rat hippocampus. *Front. Syst. Neurosci.* 7:120. 10.3389/fnsys.2013.00120 24421759 PMC3872745

[B6] Deadwyler S. A. Hampson R. E. Song D. Opris I. Gerhardt G. A. Marmarelis V. Z. (2017). A cognitive prosthesis for memory facilitation by closed-loop functional ensemble stimulation of hippocampal neurons in primate brain. *Exp. Neurol.* 287 452–460. 10.1016/j.expneurol.2016.05.031 27233622 PMC5633045

[B7] Ezzyat Y. Wanda P. A. Levy D. F. Kadel A. Aka A. Pedisich I. (2018). Closed-loop stimulation of temporal cortex rescues functional networks and improves memory. *Nat. Commun.* 9:365. 10.1038/s41467-017-02753-0 29410414 PMC5802791

[B8] Gondard E. Teves L. Wang L. McKinnon C. Hamani C. Kalia S. K. (2019). Deep Brain Stimulation Rescues Memory and Synaptic Activity in a Rat Model of Global Ischemia. *J. Neurosci.* 39 2430–2440. 10.1523/JNEUROSCI.1222-18.2019 30696731 PMC6435818

[B9] Goyal A. Miller J. Watrous A. J. Lee S. A. Coffey T. Sperling M. R. (2018). Electrical Stimulation in Hippocampus and Entorhinal Cortex Impairs Spatial and Temporal Memory. *J. Neurosci.* 38 4471–4481. 29636396 10.1523/JNEUROSCI.3049-17.2018 PMC5943975

[B10] Hampson R. E. Pons T. P. Stanford T. R. Deadwyler S. A. (2004). Categorization in the monkey hippocampus: a possible mechanism for encoding information into memory. *Proc. Natl. Acad. Sci. U. S. A.* 101 3184–3189. 14978264 10.1073/pnas.0400162101 PMC365764

[B11] Hampson R. E. Roeder B. M. Dakos A. S. She X. Moore B. Song D. (2019). Decoding memory - Memory facilitation of category information for up to 75 minutes using hippocampal stimulation via a memory decoding model. *Soc. Neurosci. Abstr.* 2019:698.604.

[B12] Hampson R. E. Roeder B. M. Johnson C. A. Dakos A. S. She X. Song D. (2018a). Facilitating memory: individualized prosthetic stimulation for memory categories. *Soc. Neurosci. Abstr.* 2018:335.326.

[B13] Hampson R. E. Simeral J. D. Deadwyler S. A. (1999). Distribution of Spatial and Nonspatial Information in Dorsal Hippocampus. *Nature* 402 610–614. 10604466 10.1038/45154

[B14] Hampson R. E. Simeral J. D. Deadwyler S. A. (2005). “Cognitive processes in replacement brain parts: A code for all reasons,” in *Toward Replacement Parts for the Brain. Implantable Biomimetic Electronics as Neural Prosthesis*, eds Berger T. W. Glanzman D. L. (Cambridge, MA: MIT Press), 111–128.

[B15] Hampson R. E. Song D. Opris I. Santos L. M. Shin D. C. Gerhardt G. A. (2013). Facilitation of memory encoding in primate hippocampus by a neuroprosthesis that promotes task-specific neural firing. *J. Neural. Eng.* 10:066013. 10.1088/1741-2560/10/6/066013 24216292 PMC3919468

[B16] Hampson R. E. Song D. Robinson B. S. Fetterhoff D. Dakos A. S. Roeder B. M. (2018b). Developing a hippocampal neural prosthetic to facilitate human memory encoding and recall. *J. Neural. Eng.* 15:036014. 10.1088/1741-2552/aaaed7 PMC6576290 29589592

[B17] Jacobs J. Miller J. Lee S. A. Coffey T. Watrous A. J. Sperling M. R. (2016). Direct Electrical Stimulation of the Human Entorhinal Region and Hippocampus Impairs Memory. *Neuron* 92 983–990. 27930911 10.1016/j.neuron.2016.10.062

[B18] Jones K. T. Johnson E. L. Berryhill M. E. (2020). Frontoparietal theta-gamma interactions track working memory enhancement with training and tDCS. *Neuroimage* 211:116615. 10.1016/j.neuroimage.2020.116615 32044440 PMC7733399

[B19] Jun S. Lee S. A. Kim J. S. Jeong W. Chung C. K. (2020). Task-dependent effects of intracranial hippocampal stimulation on human memory and hippocampal theta power. *Brain Stimul.* 13 603–613. 32289685 10.1016/j.brs.2020.01.013

[B20] Karakas S. (2020). A review of theta oscillation and its functional correlates. *Int. J. Psychophysiol.* 157 82–99. 10.1016/j.ijpsycho.2020.04.008 32428524

[B21] Kota S. Rugg M. D. Lega B. C. (2020). Hippocampal Theta Oscillations Support Successful Associative Memory Formation. *J. Neurosci.* 40 9507–9518. 10.1523/jneurosci.0767-20.2020 33158958 PMC7724134

[B22] Kragel J. E. VanHaerents S. Templer J. W. Schuele S. Rosenow J. M. Nilakantan A. S. (2020). Hippocampal theta coordinates memory processing during visual exploration. *Elife* 9:e52108. 10.7554/eLife.52108 32167468 PMC7069726

[B23] Kucewicz M. T. Berry B. M. Kremen V. Miller L. R. Khadjevand F. Ezzyat Y. (2018). Electrical Stimulation Modulates High γ Activity and Human Memory Performance. *eNeuro* 5:ENEURO.0369-17.2018. 10.1523/eneuro.0369-17.2018 29404403 PMC5797477

[B24] Larson J. Munkácsy E. (2015). Theta-burst LTP. *Brain Res.* 1621 38–50. 10.1016/j.brainres.2014.10.034 25452022 PMC4411212

[B25] Mankin E. A. Aghajan Z. M. Schuette P. Tran M. E. Tchemodanov N. Titiz A. (2021). Stimulation of the right entorhinal white matter enhances visual memory encoding in humans. *Brain Stimul.* 14 131–140. 10.1016/j.brs.2020.11.015 33279717 PMC7855810

[B26] Mankin E. A. Fried I. (2020). Modulation of Human Memory by Deep Brain Stimulation of the Entorhinal-Hippocampal Circuitry. *Neuron* 106 218–235. 10.1016/j.neuron.2020.02.024 32325058 PMC7347298

[B27] Marmarelis V. Z. Shin D. C. Song D. Hampson R. E. Deadwyler S. A. Berger T. W. (2014). On parsing the neural code in the prefrontal cortex of primates using principal dynamic modes. *J. Comput. Neurosci.* 36 321–337. 10.1007/s10827-013-0475-3 23929124 PMC3918491

[B28] Mohan U. R. Watrous A. J. Miller J. F. Lega B. C. Sperling M. R. Worrell G. A. (2020). The effects of direct brain stimulation in humans depend on frequency, amplitude, and white-matter proximity. *Brain Stimul.* 13 1183–1195. 10.1016/j.brs.2020.05.009 32446925 PMC7494653

[B29] Neter J. Wasserman W. (1974). *Applied Linear Statistical Models.* Homewood, IL: Richard D. Irwin, Inc.

[B30] Nicolas B. Sala-Padro J. Cucurell D. Santurino M. Falip M. Fuentemilla L. (2021). Theta rhythm supports hippocampus-dependent integrative encoding in schematic/semantic memory networks. *Neuroimage* 226:117558. 10.1016/j.neuroimage.2020.117558 33246130

[B31] Opris I. Santos L. M. Gerhardt G. A. Song D. Berger T. W. Hampson R. E. (2015). Distributed encoding of spatial and object categories in primate hippocampal microcircuits. *Front. Neurosci.* 9:317. 10.3389/fnins.2015.00317 26500473 PMC4594006

[B32] Reinhart R. M. G. Nguyen J. A. (2019). Working memory revived in older adults by synchronizing rhythmic brain circuits. *Nat. Neurosci.* 22 820–827. 10.1038/s41593-019-0371-x 30962628 PMC6486414

[B33] Roeder B. M. (2021a). *Chapter 2: Developing a Hippocampal Neural Prosthetic to Facilitate Human Memory Encoding and Recall of Stimulus Features and Categories.* Ph.D.thesis. North Carolina: Wake Forest University Graduate School of Arts and Sciences.

[B34] Roeder B. M. (2021b). *Chapter 3: Shared Memory Codes for Specific Information Content Across Subjects Facilitate Encoding and Recall of Stimulus Features and Categories.* Ph.D.thesis. North Carolina: Wake Forest University Graduate School of Arts and Sciences.

[B35] She X. Berger T. W. Song D. (2021). A Double-Layer Multi-Resolution Classification Model for Decoding Spatiotemporal Patterns of Spikes With Small Sample Size. *Neural. Comput.* 34 219–254. 10.1162/neco_a_01459 34758485 PMC9470026

[B36] She X. Robinson B. Flynn G. Berger T. W. Song D. (2022). Accelerating input-output model estimation with parallel computing for testing hippocampal memory prostheses in human. *J. Neurosci. Methods* 370:109492. 10.1016/j.jneumeth.2022.109492 35104492

[B37] Song D. Chan R. H. Marmarelis V. Z. Hampson R. E. Deadwyler S. A. Berger T. W. (2009). Nonlinear modeling of neural population dynamics for hippocampal prostheses. *Neural. Netw.* 22 1340–1351. 19501484 10.1016/j.neunet.2009.05.004 PMC2821165

[B38] Song D. Hampson R. E. Robinson B. S. Marmarelis V. Z. Deadwyler S. A. Berger T. W. (2016). Decoding memory features from hippocampal spiking activities using sparse classification models. *Conf. Proc. IEEE Eng. Med. Biol. Soc.* 2016 1620–1623. 10.1109/EMBC.2016.7591023 28268639

[B39] Song D. Robinson B. S. Hampson R. E. Marmarelis V. Z. Deadwyler S. A. Berger T. W. (2018). Sparse Large-Scale Nonlinear Dynamical Modeling of Human Hippocampus for Memory Prostheses. *IEEE Trans. Neural. Syst. Rehabil. Eng.* 26 272–280. 10.1109/TNSRE.2016.2604423 28113595 PMC5623111

[B40] Tambini A. Nee D. E. D’Esposito M. (2018). Hippocampal-targeted Theta-burst Stimulation Enhances Associative Memory Formation. *J. Cogn. Neurosci.* 30 1452–1472. 10.1162/jocn_a_01300 29916791 PMC7467684

[B41] Titiz A. S. Hill M. R. H. Mankin E. A. Aghajan M. Z. Eliashiv D. Tchemodanov N. (2017). Theta-burst microstimulation in the human entorhinal area improves memory specificity. *Elife* 6:e29515. 10.7554/eLife.29515 29063831 PMC5655155

[B42] Tsanov M. Manahan-Vaughan D. (2009). Long-term plasticity is proportional to theta-activity. *PLoS One* 4:e5850. 10.1371/journal.pone.0005850 19513114 PMC2688745

