# Corrigendum: Patterned hippocampal stimulation facilitates memory in patients with a history of head impact and/or brain injury

**DOI:** 10.3389/fnhum.2022.1039221

**Published:** 2022-10-06

**Authors:** Brent M. Roeder, Mitchell R. Riley, Xiwei She, Alexander S. Dakos, Brian S. Robinson, Bryan J. Moore, Daniel E. Couture, Adrian W. Laxton, Gautam Popli, Heidi M. Munger Clary, Maria Sam, Christi Heck, George Nune, Brian Lee, Charles Liu, Susan Shaw, Hui Gong, Vasilis Z. Marmarelis, Theodore W. Berger, Sam A. Deadwyler, Dong Song, Robert E. Hampson

**Affiliations:** ^1^Department of Physiology and Pharmacology, Wake Forest School of Medicine, Winston-Salem, NC, United States; ^2^Department Biomedical Engineering, Viterbi School of Engineering, University of Southern California, Los Angeles, CA, United States; ^3^Department of Neurosurgery, Wake Forest School of Medicine/Atrium Health Wake Forest Baptist, Winston-Salem, NC, United States; ^4^Department of Neurology, Wake Forest School of Medicine/Atrium Health Wake Forest Baptist, Winston-Salem, NC, United States; ^5^Department of Neurology, W. M. Keck School of Medicine, University of Southern California, Los Angeles, CA, United States; ^6^Department of Neurosurgery, W. M. Keck School of Medicine, University of Southern California, Los Angeles, CA, United States; ^7^Department of Neurology, Rancho Los Amigos National Rehabilitation Hospital, Los Angeles, CA, United States

**Keywords:** deep brain stimulation, hippocampus, memory, non-linear dynamics, traumatic brain injury, epilepsy, memory encoding, memory decoding

In the published article, an author name was incorrectly written as “Heidi M. Clary.” The correct spelling is “Heidi M. Munger Clary.”

In the published article, there was an error in the **Author contributions**. Heidi M. Munger Clary's initials were provided as “HC.” The correct initials are “HMC.”

The authors apologize for this error and state that this does not change the scientific conclusions of the article in any way. The original article has been updated.

## Publisher's note

All claims expressed in this article are solely those of the authors and do not necessarily represent those of their affiliated organizations, or those of the publisher, the editors and the reviewers. Any product that may be evaluated in this article, or claim that may be made by its manufacturer, is not guaranteed or endorsed by the publisher.

