# Supplementary material for: Patterned Hippocampal Stimulation Facilitates Memory in Patients With a History of Head Impact and/or Brain Injury
Source: Front Hum Neurosci. 2022 Jul 25;16:933401. doi: 10.3389/fnhum.2022.933401 (PMC9358788; doi:10.3389/fnhum.2022.933401)
Supplement: Supplementary file 1 [file Data_Sheet_1.PDF]

Supplemental Information

Expanded demographic table (Table I-S).

								Seizure	Epilepsy Etiology				Electrode
Patient	Test Site	TBI Type	Memory	Sex	Age	MIMO	MDM	Focus in HPC	Head Injury	Genetic	Major Medical	Neuroanatomic	Implant Sites
Keck06	KHUSC	TBI	Normal	M	42	√		√ - bilat HPC	√				L. Ant.
Keck08	KHUSC	Control	Impaired	M	26	√	√	√ - L. HPC			√		Bilat. Ant.
Keck15	KHUSC	Control	Impaired	F	20		√	√ - bilat HPC					Bilat. Ant.
Rancho01	RLANRH	RMBI	Impaired	M	35		√	√ - bilat HPC					Bilat. Ant.+Post.
Rancho07	RLANRH	Control	Normal	M	35		√	√ - L. HPC					Bilat. Ant.+Post.
Wake14	WFSM	RMBI	Impaired	M	35	√		Temporal					Bilat. Ant.+Post.
Wake15	WFSM	TBI	Impaired	M	45	√		Temporal	√				Bilat. Ant.
Wake16	WFSM	Control	Normal	M	21	√		Extratemporal					Bilat. Ant.+Post.
Wake17	WFSM	TBI	Impaired	F	31	√		Temporal	√				Bilat. Ant.
Wake18	WFSM	Control	Normal	F	55	√		√ - R. HPC		√			Bilat. Ant.
Wake19	WFSM	TBI	Normal	F	33	√		√ - L. HPC	√				L. Ant.
Wake20	WFSM	Control	Normal	F	31	√	√	Extratemporal		√			R. Ant.+Post.
Wake21	WFSM	TBI	Impaired	F	26	√	√	Extratemporal				√	Bilat. Ant.
Wake22	WFSM	RMBI	Impaired	M	48		√	√ - R. HPC		√			Bilat. Ant.
Wake23	WFSM	RMBI	Normal	F	51		√	Temporal				√	R. Ant.
Wake24	WFSM	Control	Normal	F	33		√	Temporal					L. Ant.
Wake25	WFSM	Control	Impaired	F	67		√	Extratemporal			√		Bilat. Ant.
Wake26	WFSM	Control	Impaired	M	23			√ - bilat HPC					Bilat. Ant.
Wake28	WFSM	RMBI	Impaired	F	55		√	√ - L. HPC					Bilat. Ant.
Wake29	WFSM	Control	Normal	F	38		√	Temporal		√			L. Ant.
Wake30	WFSM	RMBI	Normal	M	55		√	√ - bilat HPC		√			Bilat. Ant.
Wake34	WFSM	TBI	Normal	F	40		√	√ - R. HPC			√		Bilat. Ant.
Wake35	WFSM	RMBI	Impaired	M	20	√		Extratemporal		√			Bilat. Ant.
Wake36	WFSM	TBI	Normal	M	42	√		Temporal	√				L. Ant.
Wake37	WFSM	Control	Impaired	F	41	√	√	√ - L. HPC				√	Bilat. Ant.

Subjects tested in the report. This table is expanded from Table I in main text to provide additional information regarding the epilepsy patients who were subjects in this study.

TEST SITE: KHUSC = Keck Hospital / School of Medicine, University of Southern California; RLANRH = Rancho Los Amigos National Rehabilitation Hospital; WFUSM – Wake Forest University School of Medicine (Atrium Health Wake Forest Baptist). TBI TYPE – TBI = Traumatic Brain Injury, subject has history of serious head injury (may include loss of consciousness); RMBI = Repeated Mild-Moderate Brain Injury, subject history indicates falls, sports injuries, or head impacts with no loss of consciousness; Control = no history of head impact. MEMORY - Normal = no evaluation of memory impairment in pre-surgical neuropsychological

evaluation; Impaired = pre-surgical neuropsychological evaluation included an assessment of mild-to-moderate memory impairment. MIMO = subject was tested with hippocampal stimulation derived from nonlinear Multi-Input, Multi-Output (MIMO) model of hippocampal CA1 neural ensemble activity. MDM = subject was tested with hippocampal stimulation derived from nonlinear Memory Decoding Model (MDM) of hippocampal CA1 neural ensemble activity.

Epilepsy patient information. Seizure Focus: list known identification of primary seizure focus prior to electrode implant. √ - Seizures were present in hippocampus (followed by lateralization if known). Etiology – If known, medical events associated with seizure onset. Head Injury is indicated if patient suffered first seizure after a known head impact. Genetic – no patients were definitively identified with genetically-linked epilepsy, however, indicated patients had family history or other family members with seizure disorder. Major medical – first seizure onset was associated with a medical event (e.g. infection). Neuroanatomic – patient exhibited morphological irregularities of brain anatomy. Implant sites – location of StereoEEG Macro-micro depth electrodes. Localization: L. – Left, R. – Right, bilat – Bilateral, HPC – hippocampus, Ant. – Anterior, Post. – Posterior.
